# Identifying prioritization of potential targets for idiopathic pulmonary fibrosis: Proteome-wide Mendelian randomization and colocalization analyses

**DOI:** 10.1016/j.clinsp.2026.100913

**Published:** 2026-03-18

**Authors:** Gexiang Cai, Jingjing Liu, Mengsi Cai, Lianyou Shao

**Affiliations:** Department of Respiratory and Critical Care Medicine, The First Affiliated Hospital of Wenzhou Medical University, Wenzhou, Zhejiang, China

**Keywords:** Idiopathic pulmonary fibrosis, Plasma protein, Proteome-wide mendelian randomization, Colocalization Analyses

## Abstract

**Background:**

Idiopathic Pulmonary Fibrosis (IPF) is a chronic and progressive lung disorder characterized by limited therapeutic options. Thus, identifying new therapeutic targets for IPF is imperative.

**Methods:**

The authors employed 2 large-scale circulating proteomic and IPF GWAS (Ncase = 2189, Ncontrol = 407,609) to conduct proteome-wide Mendelian randomization meta-analysis. Subsequently, the authors utilized Summary Mendelian Randomization analysis and Bayesian colocalization to enhance the screening of proteins. Lastly, MR analysis of protein-protein level ratio, two-step MR method, enrichment analysis, Protein-Protein Interaction (PPI), and druggability assessment were applied to explore the potential mechanisms of candidate proteins.

**Results:**

Through proteome-wide Mendelian randomization analysis and multiple validations, the authors identified 3 plasma proteins significantly associated with IPF. With strong evidence of colocalization and consistent support from SMR analysis, the gene-predicted MASP1 was correlated with decreased IPF risk (OR_meta_ = 0.46, 95% CI_meta_: 0.22‒0.99, P_meta_ = 0.0478, PPH4 = 0.92). With suggestive colocalization support (PPH4 = 0.50), the levels of S100A11 were inversely related to IPF risk, with odds ratios of 0.53 (95% CI_MR1_ 0.34–0.83, P_MR1_ = 0.0053). Conversely, the gene-predicted WFIKKN2 was associated with an increased risk of IPF (OR_meta_ = 1.31, 95% CI_meta_ 1.15–1.48, P_meta_ = 2.48E-05, PPH4 = 0.50). Their association was further supported by SMR analysis. Moreover, MR analysis of protein level ratio, mediator MR analysis, enrichment, and PPI network provided insights into the potential drug development based on the proteins.

**Conclusions:**

Our proteome-wide Mendelian Randomization analysis highlighted MASP1, S100A11, and WFIKKN2 as potential targets for further clinical investigation in IPF. The specific mechanisms by which these proteins influence IPF pathogenesis require further elucidation through continued research.

## Highlights

• Mendelian randomization reveals causal plasma proteins for idiopathic pulmonary fibrosis.
• MASP1 and S100A11 are novel therapeutic targets lowering IPF risk.
• WFIKKN2 is a potential therapeutic target that increases IPF risk.

## Introduction

Idiopathic Pulmonary Fibrosis (IPF) is a progressive lung disease, affecting nearly 3 million individuals worldwide and carrying a median survival time of 3.8-years post-diagnosis.^1^^,^^2^ Its complex pathophysiology, encompassing molecular, cellular, and genetic alterations, poses substantial challenges for effective therapy development. Currently, only Nintedanib and Pirfenidone are FDA-approved for IPF treatment, but they cannot cure the disease. For end-stage patients, lung transplantation may be the only hope for survival. Consequently, exploring novel therapeutic targets is essential for IPF treatment.

Plasma proteins, key blood components, often undergo concentration and activity changes during disease progression, making them promising biomarkers and therapeutic targets. In 2017, nearly 75 % of the drugs approved by the FDA were therapies targeting human proteins.^3^ Advances in genomics, proteomics, and Genome-Wide Association Studies (GWAS) have identified many genetic variants linked to plasma proteins, known as “plasma protein Quantitative Trait Loci” (pQTL). Phenome-wide Mendelian Randomization (MR), using pQTLs as Instrumental Variables (IVs) to assess causality, offers a reliable approach for drug target discovery. Compared with conventional observational studies, the MR method helps mitigate the effects of confounding factors and boosts the success rate in drug development endeavors.^4^ To date, no studies have yet combined MR methods with IPF and pQTL data to identify potential drug targets for IPF.

In this research, the authors conducted an extensive proteome-wide MR analysis to explore potential candidate targets associated with IPF.

## Methods

**Fig. 1:**
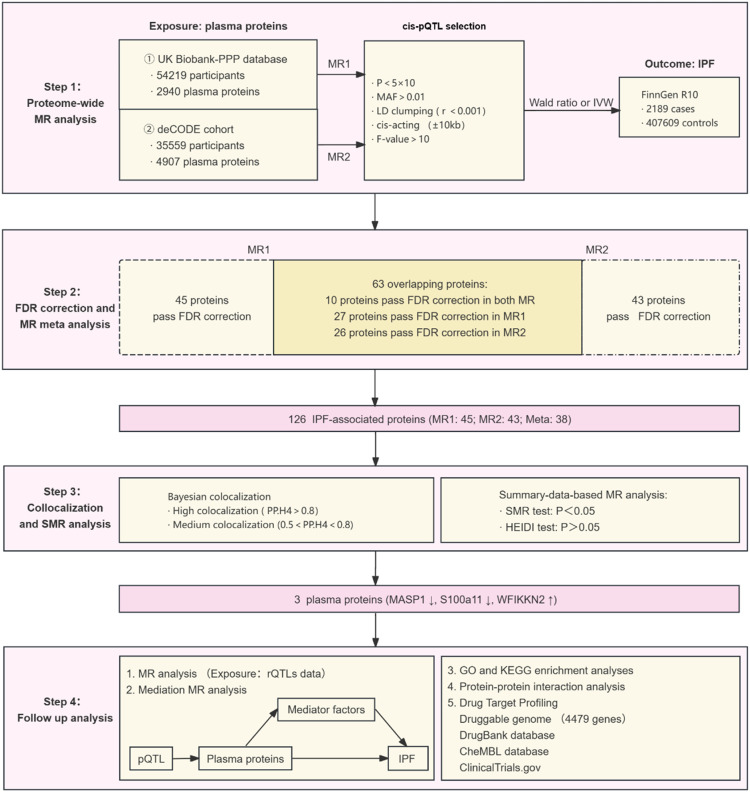
Flowchart of the study design.

In this study, all data were derived from publicly available publications and databases. The study followed the STROBE-GE guidelines, with details in Supplement Table 1. The specific study design is illustrated in Fig. 1. Flowchart of the study design.

### Proteomic and outcome data source

The authors utilized pQTL data from two large-scale studies.^5^^,^^6^ Sun et al. characterized the plasma proteome of 54,219 UK Biobank participants, identifying pQTLs for 2940 proteins.^6^ Another proteomics dataset came from the deCODE Genetics team, which included 35,559 Icelanders, examining genetic associations for 4907 circulating protein levels.^5^ For our MR analysis, pQTL selection criteria were: (i) Single Nucleotide Polymorphisms (SNPs) located within ±1 Mb of the gene region (cis-acting pQTLs); (ii) A genome-wide significant association (p-value < 5 × 10^−8^); (iii) SNPs and proteins outside the major histocompatibility complex region (Chromosome 6: 25.5–34.0 Mb); and (iv) A Linkage Disequilibrium (LD) cluster r2 value less than 0.001.

The GWAS summary data for IPF were derived from the FinnGen R10 consortium (https://r10.finngen.fi/). The dataset comprises information from 2189 IPF patients and 407,609 control individuals. The GWAS data utilized in this research were obtained from independent, non-overlapping samples of European ancestry (Supplement Table 2).

### Proteome-wide MR analysis

The authors initially conducted a two-sample MR analysis with plasma proteins as exposure and IPF as outcome. For MR1 analysis, exposure data from the UK Biobank Plasma Proteome Project (UKB-PPP); for MR2 analysis, data from the deCODE Genetics study (Fig. 1.). Proteins with one SNP used the Wald ratio method. For those with two or more SNPs, the authors employed the Inverse-Variance Weighted method (IVW), MR Egger, weighted median method, and weighted mode method to assess causality, with IVW being the primary method.^7^ Then the authors adjusted raw p-values with Benjamini-Hochberg False-Discovery Rate (FDR), considering *p* < 0.05 significant.^8^ Results were reported as Odds Ratios (OR) with 95 % Confidence Intervals (95 % CI). The meta-analysis was performed on proteins that were significant after FDR correction in MR1 and MR2 analyses. The primary significance threshold for the meta-analysis was a nominal *p* < 0.05, as this step was intended for effect estimation of pre-qualified candidates. However, FDR-corrected p-values for the meta-analysis are also provided in the supplement. (Fig. 1). The heterogeneity was assessed using *I^2^* statistic to determine whether to use random-effects or fixed-effects models.

Methods included MR-Egger regression for horizontal pleiotropy; and Cochran’s *Q* test for heterogeneity.^9^ Steiger filtering checked if SNPs correlated more strongly with the outcome than the exposure variable, with non-passing SNPs excluded from analysis.

### Bayesian colocalization and SMR analysis

Bayesian collocation analysis was used to determine whether proteins with positive MR results share causal genetic variants with IPF. To broadly capture proteins of potential interest, the authors included all proteins identified in the initial Proteome-wide MR analysis in the colocalization analysis. The Posterior Probability of Hypothesis-4 (PPH4: signifies that the SNP within the selected locus is concurrently associated with both traits, and is a shared SNP) greater than 0.8 is considered strong evidence of colocalization. Moderate colocalization is defined as a PPH4 ranging from 0.5 to 0.8.

Candidates showing Colocalization evidence were then advanced to Summary-data-base Mendelian Randomization (SMR) analysis and Heterogeneity in Dependent Instruments (HEIDI) testing. The publicly available eQTL data from either eQTLGen (https://www.eqtlgen.org/) or the Genotype-Tissue Expression (GTEx-V8) project. Cis-eQTLs refer to genetic variations significantly linked to the expression of specific genes affected by medications, with a p-value lower than 5 × 10^–8^. When there are three or more SNPs present, the HEIDI test determines if the observed association with a disease phenotype is due to shared genetic variation rather than genetic linkage. A p-value greater than 0.05 from the HEIDI test suggests a robust causal relationship, unlikely influenced by genetic linkage.

Proteins were prioritized as putative causal targets only if they demonstrated evidence of co-localization. Additionally, they were required to show a significant causal effect in the SMR analysis (P_SMR_ < 0.05) without significant heterogeneity in the HEIDI test (P_HEIDI_ > 0.05).

### Statistical power consideration

The authors also used the online tool mRnd (http://cnsgenomics.com/shiny/mRnd/) to calculate statistical power. Input parameters for power calculations in this tool include sample size, Type I error rate, proportion of cases in the study, odds ratio, and proportion of variance explained for the association between the SNP. Presently, there is no standardized methodology for calculating a unique *R^2^* value for a meta-analyzed Mendelian randomization estimate. Consequently, the authors performed statistical power calculations solely based on the results from the initial proteome-wide MR analysis. A statistical power greater than 0.8 typically indicates that a study has a high detection capability, effectively countering the impact of sampling error and random variation, and reducing the risk of Type II errors (i.e., false negatives).^10^

### Pathway and functional enrichment analysis

The authors conducted Gene Ontology (GO) and Kyoto Encyclopedia of Genes and Genomes (KEGG) enrichment analyses to deepen our understanding of the biological functions and metabolic pathways of similarly expressed proteins. GO analysis assesses gene enrichment in biological processes, molecular functions, and cellular components compared to a reference genome. KEGG enrichment analysis focuses on identifying the collaborative roles of genes within specific metabolic pathways.

### Protein-protein ratios MR analysis

Plasma proteins form a complex network. To explore the biological mechanisms of druggable proteins, the authors used the ratios between protein levels to explore the association between protein-protein interactions and IPF. The ratios QTLs (rQTLs) GWAS data came from the research of Karsten Suhre.^11^ Karsten Suhre et al. used Olink proteomics data for 1463 proteins measured in 52,705 UK Biobank samples, identifying 4248 associations with 2821 protein level ratios. The Cis-rQTLs were selected based on the previously mentioned criteria.

### Mediation MR analysis

Previous MR studies have implicated Body Mass Index (BMI),^12^ smoking initiation,^13^ telomere length,^12^ hypothyroidism,^14^ C-reactive protein,^15^ and lung function^16^ have a causal association with IPF. The authors conducted a two-step MR approach for mediation analysis to measure the influence of identified proteins on IPF through these risk factors. Detailed information on the GWAS summary data for risk factors is presented in Supplement Table 2. The total effect of the exposure on the outcome can be decomposed into direct and indirect effects.^17^ The direct effect, calculated via primary MR analysis, reflects the immediate impact of proteins on IPF. The indirect effect, estimated using the product method, shows the proteins' influence through mediators. Standard errors and confidence intervals were calculated using the delta method.^17^

### Druggable proteins identification

To explore the potential candidate targets and the interactions in IPF, the authors conducted a Protein-Protein Interaction (PPI) analysis on identified plasma proteins and previously recognized drug targets for IPF using the STRING database (https://string-db.org) with a minimum required interaction score of 0.4.

The authors also assessed the druggability of candidate proteins by referencing Finan's study on 4479 druggable genes,^18^ the DrugBank database, the ChEMBL database (https://www.ebi.ac.uk/chembl), and ClinicalTrials (https://www.ClinicalTrials.gov). These 4479 druggable genes are stratified into three tiers: Tier 1 includes targets of marketed drugs and clinical candidates; Tier 2 comprises targets closely related to marketed drug targets or drug-like compounds; and Tier 3 encompasses targets with more distant similarities to marketed drug targets. Other databases offered details on the drugs they are involved and provided information on the clinical development stages of the target proteins.

## Results

### Proteome-wide MR analysis and colocalization

**Fig. 2:**
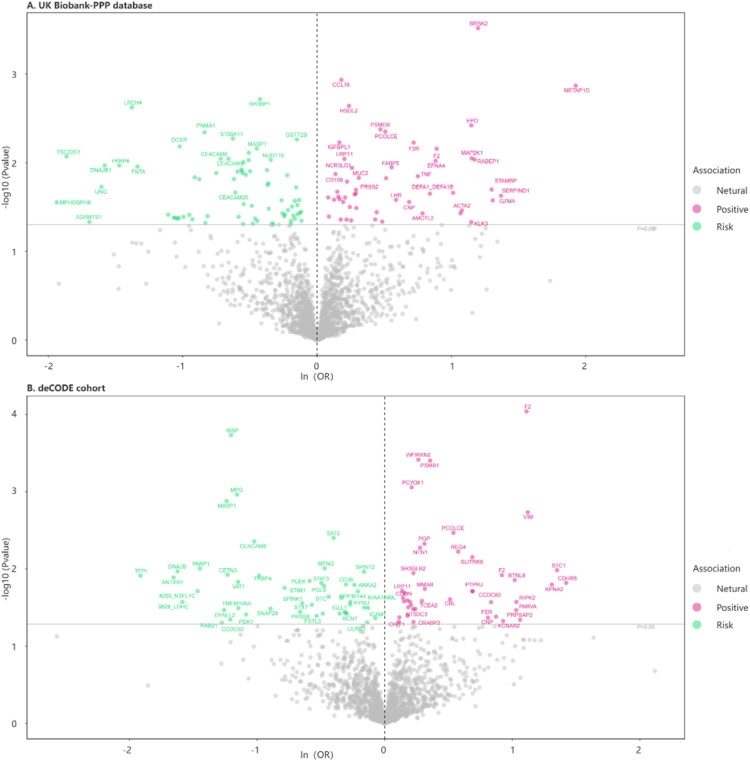
Volcano plot of the MR results for plasma proteins on IPF. (A) UK Biobank-PPP database; (B) deCODE cohort.

In MR1 analysis, 1908 plasma proteins with available pQTLs from the UKB-PPP eQTL database were identified. After FDR correction, 82 plasma proteins were found to have a causal relationship with IPF (Fig. 2, Supplement Table 3). Among these, 35 proteins were associated with an increased IPF risk, while 47 were associated with a decreased risk. In MR2 analysis, using the deCODE Genetics database containing pQTLs for 1675 plasma proteins, 79 plasma proteins were identified to have a causal link with IPF after FDR correction (Fig. 2, Supplement Table 4). Within this group, 41 proteins were associated with an increased IPF risk, while 38 were associated with a decreased risk. All proteins remained directionally concordant using the MR Egger, weighted median method, and weighted mode method. By intersecting the results from two MR analyses, 63 plasma proteins were identified for subsequent MR meta-analysis (Fig. 1). Through a meta-analysis of the IVW results, it was further confirmed 38 proteins have a causal relationship with IPF (Supplement Table 5).

Some proteins lacked sufficient SNPs to perform Cochran's *Q* test and the MR Egger intercept test due to the exclusive use of cis-pQTL as IVs. For proteins with an adequate number of SNPs, no horizontal pleiotropy was found (Supplement Table 6). However, heterogeneity was detected in the MR estimates for IGDCC4, S100A12, SAT2, STX7, and TFPI (*p* < 0.05 derived from Cochran's *Q* test) (Supplement Table 6). A random-effects IVW model was used to address this heterogeneity. Steiger filtering confirmed no reverse causation bias affected the MR estimates, and all F-statistics exceeded 20 (Supplement Table 7‒8).

**Fig. 3:**
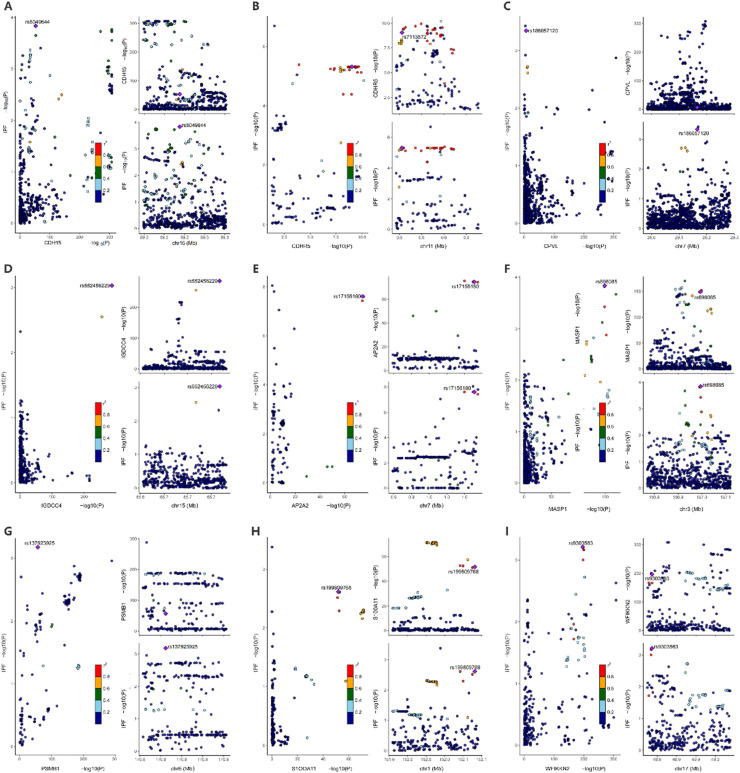
The results of colocalization analysis. (A) CDH15; (B) CDHR5; (C) CPVL; (D) IGDCC4; (E) AP2A2; (F) MASP1; (G) PSMB1; (H) S100A11; (I) WFIKKN2.

**Fig. 4:**
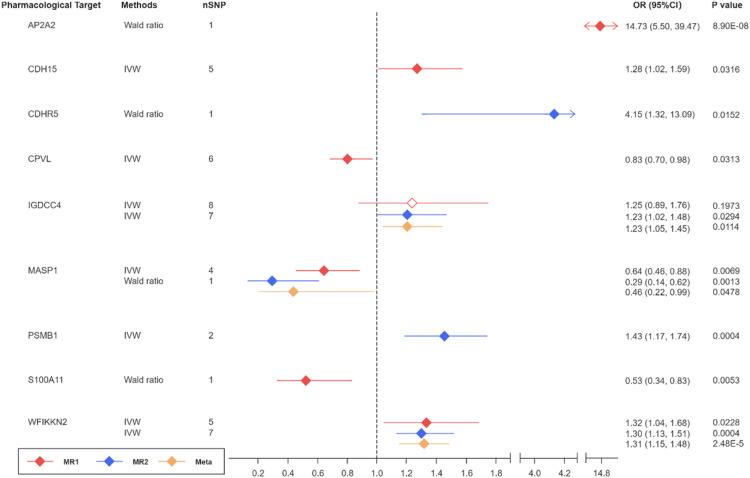
Causal effects of plasma proteins on IPF through MR and meta-analysis. 6 proteins could increase IPF risk: AP2A2 (OR_MR1_ = 14.73, 95 % CI _MR1_: 5.50–39.47, P_MR1_ = 8.90 × 10^–8^), CDH15 (OR_MR1_ = 1.28, 95 % CI_MR1_: 1.02–1.59, P_MR1_ = 0.0316), CDHR5 (OR_MR2_ = 4.15, 95 % CI_MR2_: 1.32–13.09, P_MR2_ = 0.0152), IGDCC4 (OR_meta_ = 1.23, 95 % CI_meta_: 1.05–1.45, P_meta_ = 0.0114), PSMB1 (OR_MR2_ = 1.43, 95 % CI_MR2_: 1.17–11.74, P_MR2_ = 0.0004), and WFIKKN2 (OR_meta_ = 1.31, 95 % CI_meta_: 1.15–1.48, P_meta_ = 2.48 × 10^–5^); and 3 proteins could decrease its risk: CPVL (OR_MR1_ = 0.83, 95 % CI_MR1_: 0.70–0.98, P_MR1_ = 0.0313), MASP1 (OR_meta_ = 0.46, 95 % CI_meta_: 0.22–0.99, P_meta_ = 0.0478) and S100A11 (OR _MR1_ = 0.53, 95 % CI _MR1_: 0.34–0.83, P_MR1_ = 0.0053).

Then, the authors conducted a colocalization analysis on 126 proteins previously mentioned. Bayesian colocalization analysis strongly suggested that AP2A2 (PPH4 = 0.99), CDHR5 (PPH4 = 0.93), MASP1 (PPH4 = 0.92), and CDH15 (PPH4 = 0.91) shared the same variant with IPF (Fig. 3, Supplement Table 9). Additionally, moderate support for colocalization was observed for 5 proteins: CPVL (PPH4 = 0.74), IGDCC4 (PPH4 = 0.54), PSMB1 (PPH4 = 0.53). Additionally, suggestive evidence for colocalization was observed for WFIKKN2 (PPH4 = 0.50), and S100A11 (PPH4 = 0.50) (Fig. 3). These proteins were carried forward for further validation. The MR analyses of the effect of these 9 proteins on IPF are depicted in Fig. 4.

For five proteins ‒ BRSK2, MUC2, LSP1, EFNA1, and FAM13A ‒ their association with IPF may be influenced by two distinct causal variants (PPH3 > 0.8). Most other protein-outcome pairs exhibited limited evidence of colocalization (Supplement Table 9).

### SMR analysis and HEIDI test

The authors performed SMR analyses and the HEIDI test to verify findings. Among the 9 proteins examined: MASP1, PSMB1, S100A11, and WFIKKN2 passed both tests (P_SMR_ < 0.05, P_HEIDI_ > 0.05) (Supplement Table 10). Consistent with the MR analysis results, WFIKKN2 expression increased IPF risk (OR = 1.16, 95 % CI: 1.01–1.35, *p* = 0.0404). MASP1 and S100A11 expression decreased IPF risk (OR = 0.57, 95 % CI: 0.42–0.77, *p* = 0.0002; OR = 0.53, 95 % CI: 0.34–0.83, *p* = 0.0059) (Supplement Table 10). Interestingly, PSMB1 gene expression had an opposite effect compared to its plasma protein, so it was excluded. CPVL and IGDCC4 did not pass SMR test, and CDH15 and CDHR5 did not pass the HEIDI test.

Guided by MR analysis, colocalization analysis, SMR validation, and HEIDI tests, the authors have ultimately identified 3 proteins (MASP1, S100A11, and WFIKKN2) as the most promising drug targets for IPF (Table 1).

**Table 1:** Evidence supporting potential proteins for which expression was significantly associated with IPF.

**Protein**	**MR analysis**			**Steiger filtering**	**Colocalization**	**SMR analysis**	**HEIDI test**
	**MR Analysis**	**OR (95 % CI)**	**p-value**				
AP2A2	MR2	14.73 (5.50, 39.47)	8.90E-08	√	++	√	×
CDH15	MR1	1.28 (1.02, 1.59)	0.0316	√	++	√	×
CDHR5	MR2	4.15 (1.32, 13.09)	0.0152	√	++	√	×
CPVL	MR1	0.83 (0.70, 0.98)	0.0313	√	+	×	√
IGDCC4	Meta	1.23 (1.05, 1.45)	0.0114	√	+	×	√
MASP1	Meta	0.46 (0.22, 0.99)	0.0478	√	++	√	√
PSMB1	MR2	1.43 (1.17, 1.74)	0.0004	√	+	√^a^	√
S100A11	MR1	0.53 (0.34, 0.83)	0.0053	√	+	√	√
WFIKKN2	Meta	1.31 (1.15, 1.48)	2.5E-05	√	+	√	√

A check mark denotes pass; a cross mark denotes fail; a plus sign indicates moderate strength colocalization; two plus signs indicate high strength colocalization; IPF, Idiopathic Pulmonary Fibrosis; MR, Mendelian Randomization; OR, Odds Ratios; 95 % CI, 95 % Confidence Intervals; SMR, Summary-data-based Mendelian Randomization.

a PSMB1 gene expression had an opposite effect compared to its plasma protein, so it was excluded.

### Statistical power consideration

Post-hoc power calculations demonstrated high and consistent power for WFIKKN2 (MR1 = 0.95; MR2 = 0.96), consequently affording high confidence in its causal role. Conversely, power for MASP1 was highly variable between datasets (MR1 = 0.77; MR2 = 0.48), highlighting the value of meta-analysis in integrating these disparate estimates (Supplement Table 11). The association for S100A11 was supported by more limited power (MR1 = 0.53), suggesting that while indicative of a true effect, the precision of the estimate is constrained and warrants future replication in larger studies.

### Exploring the biological context of identified proteins

**Fig. 5:**
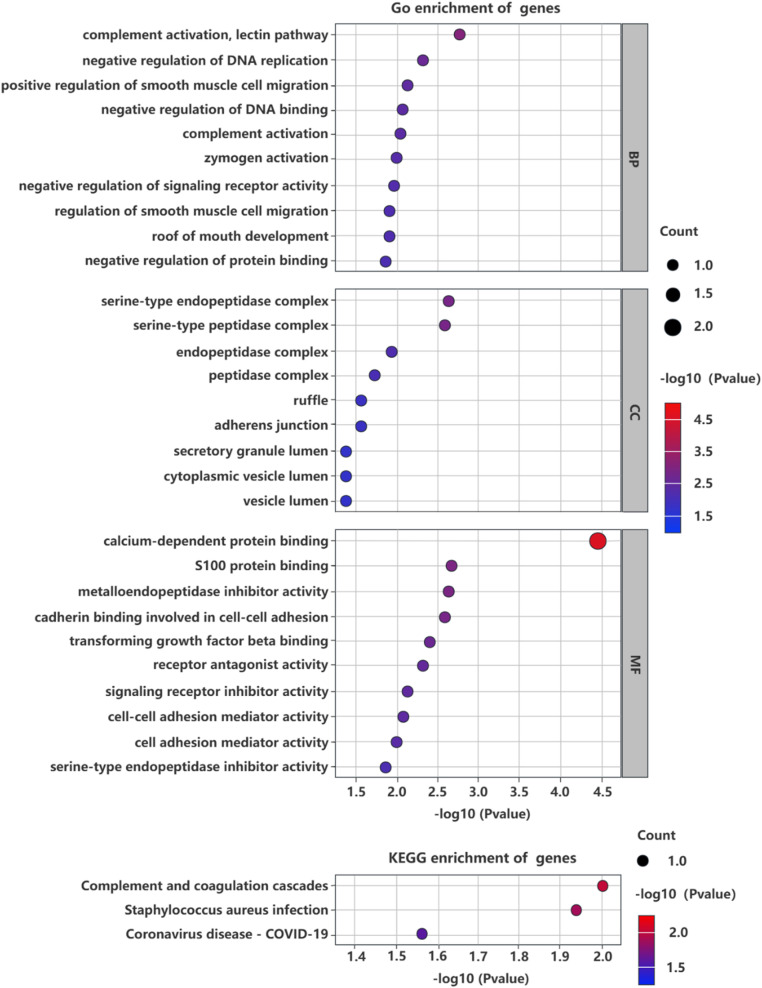
GO and KEGG enrichment analysis of 3 identified proteins for treatment of IPF.

To contextualize the biological roles of the three candidate proteins, the authors examined their GO annotations and KEGG pathways. GO annotations revealed that biological process terms were predominantly enriched in complement activation, smooth muscle cell migration, negative regulation of DNA replication or DNA binding (Fig. 5). In terms of cellular components, the terms were mainly enriched in serine‑type endopeptidase complex, serine‑type peptidase complex, adherens junction, and ruffle. Moreover, molecular function was primarily associated with calcium-dependent protein binding, S100 protein binding, and cadherin binding involved in cell-cell adhesion. KEGG analysis indicated these proteins are involved in complement and coagulation cascades, staphylococcus aureus infection, and Coronavirus Disease ‒ COVID-19 pathways (Fig. 5).

### Protein-protein ratios MR analysis

For the 3 most potential drug targets, the authors obtained rQTLs data for S100A11 ratios with 6 other proteins. No rQTLs data were available for MASP1 and WFIKKN2 ratios. The IVW method indicated that NCF2/S100A11 ratio had a causal relationship with IPF (OR = 0.62, 95 % CI: 0.40‒0.97, *p* = 0.0.382) (Supplement Table 11 and 12). No causal associations were found for the other five protein ratios. Sensitivity analyses found no directional pleiotropies (st Table 13).

### Mediation MR analysis

Using a two-sample MR approach, the authors identified 9 factors causally associated with IPF, including body fat percentage, BMI, Forced Vital Capacity (FVC), Forced Expiratory Volume in one second (FEV1), hypothyroidism, age of smoking initiation, trunk fat mass, and whole-body fat mass (Supplement Table 14). No evidence of horizontal pleiotropy was found (Supplement Table 15).

Mediated MR analysis linked WFIKKN2 to lung function decline. Specifically, a higher WFIKKN2 level was associated with increased risk of FVC decline (β = 0.0075, 95 % CI = 2.4e-05 to 0.0151), mediating IPF onset with a mediation effect of 2.69 % (95 % CI = 0.01 % to 5.39 %, *p* = 0.04428) (Supplement Table 16). Similarly, elevated WFIKKN2 level was also associated with increased risk of FEV1 decline (β = 0.0086, 95 % CI = 0.0010 to 0.0162), with a mediation effect of 3.07 % (95 % CI = 0.36 % to 5.78 %, *p* = 0.0232) (Supplement Table 16).

### Druggability of identified proteins

**Fig. 6:**
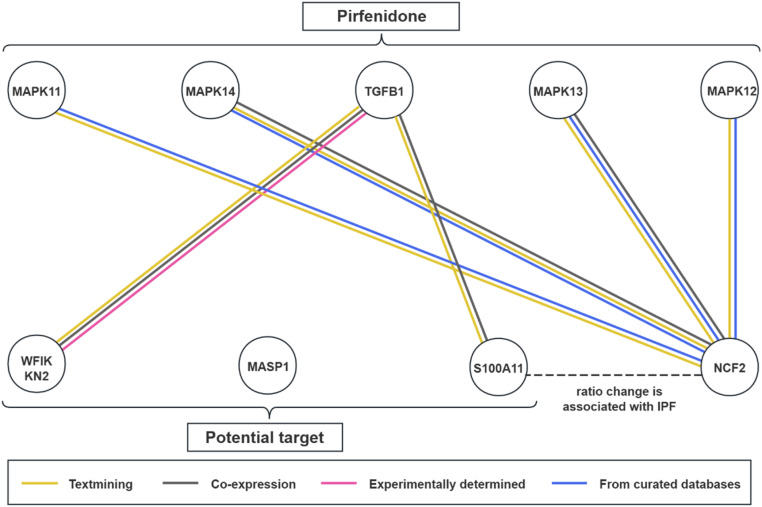
Interaction between identified plasma proteins and current medication targets for IPF.

MASP1 and WFIKKN2 are listed in the druggable gene list at Tier 3. Ivacaftor, as a selective MASP1 inhibitor, can be used to treat cystic fibrosis and prostate cancer. Emtricitabine/Tenofovir, recognized as MASP1 inhibitors, is utilized in the management of immunodeficiency conditions. OMS-906, as a novel MASP1 inhibitor, currently has 4 ongoing clinical trials recruiting participants to evaluate its efficacy in C3 glomerulopathy and paroxysmal nocturnal hemoglobinuria (Supplement Table 17). S100A11 is not yet recognized as a potential drug target. However, the PPI network indicates that S100A11 and WFIKKN2 share co-expression and other relationships with TGFB, a target of pirfenidone, suggesting promising druggability (Fig. 6). No connections were identified between the potential protein targets and the therapeutic target of Nintedanib.

## Discussion

Our study integrates plasma protein-centric multi-omics to identify potential therapeutic targets for IPF. Genetically predicted MASP1 and S100A11 are protective against IPF, while higher WFIKKN2 levels are associated with increased IPF risk. These findings provide genetic evidence supporting further investigation. Additionally, through intermediary MR analysis, enrichment analysis, druggability assessment of proteins, and PPI network analysis, we've deepened our understanding of these proteins' mechanisms and their potential in drug development.

Although our study does not elucidate the precise mechanism, the established biological functions of MASP1 allow us to hypothesize its potential role in IPF. MASP1, mannan-binding lectin serine protease 1, is a serine protease enzyme involved in various physiological processes.^19^ It has been observed that MASP1 has synergistic effects with other pro-inflammatory mediators, enhancing each other's roles in regulating IL-8.^20^ Specifically, the combination of MASP1 and IFNγ has been shown to increase IL-8 expression, which is associated with pulmonary neutrophil infiltration during IPF.^21^ MASP1′s ability to induce pro-inflammatory activation of endothelial cells is also noteworthy,^20^ as endothelial cell abnormalities and the dysregulation of endothelial protective pathways play a significant role in IPF pathogenesis.^22^ This may suggest a potential link between MASP1 and IPF, although the precise mechanisms warrant further investigation.

S100A11, a member of the S100 protein family, is widely expressed in human tissues, including the lungs, and is involved in regulating enzyme activity, cell growth, apoptosis induction, and inflammatory responses.^23^ Despite limited research in the lung field, emerging evidence points to a possible role for S100A11 in respiratory diseases. A study by Mi Cheng et al. showed that S100A11 mitigates airway hyperresponsiveness in rats by relaxing over-contracted airway smooth muscles.^24^ Additionally, research by Qiuyan Liang et al. indicated that S100A11 enhances M2a macrophage activation and pulmonary inflammation in asthma models.^25^ Considering the role of S100A11 in pulmonary inflammatory responses, future research could investigate the hypothesized role of S100A11 in the pathogenesis of IPF.

WFIKKN2 is a multi-domain protein that encompasses a whey acidic protein, follistatin, an immunoglobulin, two Kunitz-type protease inhibitor domains, and a netrin domain. Recent studies have uncovered interactions between WFIKKN proteins and various members of the TGFB superfamily, which play a pivotal role in fibrogenesis.^2^ Our mediation MR analysis found a small but statistically significant proportion of the total effect of WFIKKN2 on IPF risk was mediated through FVC (2.69 %) and FEV1 (3.07 %). Although the mediated proportions were modest, the identification of lung function as a downstream pathway offers a testable mechanistic hypothesis for future functional studies of WFIKKN2.

Our MR analysis nominates three potential proteins for IPF, yet these findings require careful contextualization. First, a major challenge in Mendelian randomization lies in interpreting causal estimates characterized by wide confidence intervals, as illustrated by MASP1 in our meta-analysis. Although such imprecision limits the reliability of estimating the exact magnitude of the effect, it does not necessarily invalidate the existence of a causal relationship. Under these circumstances, the convergence of evidence derived from orthogonal genetic methodologies becomes essential. Here, convergence of evidence from orthogonal methods becomes essential. The strong colocalization signal (PPH4 > 0.9) for MASP1 substantially reduces the likelihood of confounding by linkage disequilibrium, while validation via SMR provides complementary support. Thus, the collective weight of nominally significant MR, robust colocalization, and significant SMR findings strengthens the causal hypothesis for MASP1, despite statistical imprecision in the MR estimate alone.

Secondly, our findings also illustrate that the utility of meta-analysis in Mendelian randomization is context-dependent, extending beyond mere power enhancement. The contrast between WFIKKN2 and MASP1 is particularly informative. WFIKKN2 demonstrates the ideal scenario: consistent effects and strong instruments across datasets allowed the meta-analysis to produce a high-precision, high-power estimate, robustly supporting its causal role. However, MASP1 represents a more complex case. Here, the meta-analysis served not to amplify power, but to integrate heterogeneous estimates and instrument strengths from the UKB-PPP and deCODE datasets. Consequently, the pooled estimate, while more balanced, is appropriately accompanied by a wide confidence interval that honestly reflects the underlying variability. Thirdly, the statistical power of our MR analysis was limited by the sample size of the available IPF GWAS. While this was sufficient to identify targets with moderate to large effect sizes, such as WFIKKN2, it resulted in imprecise estimates for others, like MASP1, and means the authors cannot rule out causal roles for many proteins with more subtle effects. Future studies with larger IPF GWAS sample sizes will be essential to expand the map of potential therapeutic targets.

Nevertheless, it is important to acknowledge the limitations of genetic evidence in directly guiding drug development. Our study identifies proteins with a causal, likely protective (MASP1, S100A11) or detrimental (WFIKKN2) role in IPF. For protective proteins, therapeutic mimicry of their effects would require agonist drugs, which are considerably more challenging to develop than the antagonists used to inhibit detrimental proteins. Consequently, while WFIKKN2 represents a conventional drug target, advancing MASP1 or S100A11 would necessitate innovative therapeutic modalities. Furthermore, human genetic evidence primarily de-risks target engagement and serves as a starting point; crucially, successful drug development depends on extensive subsequent validation in IPF models to confirm efficacy and safety.

## Conclusions

This study utilized an integrative genetic approach to identify associations between circulating proteins and IPF risk. By integrating GWAS data, the proteins WFIKKN2, MASP1, and S100A11 were identified as potential therapeutic targets for IPF. These targets require further experimental validation to assess their applicability in IPF treatment.

## Ethics approval statement

No participants were directly engaged in the overall progression of our investigation.

## Funding

No funding was received for this study.

## Clinical trial number

Not applicable.

## Abbreviations

IPF, Idiopathic Pulmonary Fibrosis; MR, Mendelian Randomization; GWAS, Genome-Wide Association Study; SMR, Summary-data-based Mendelian Randomization; pQTL, Plasma protein Quantitative Trait Loci; IVs, Instrumental Variables; SNPs, Single Nucleotide Polymorphisms; UKB-PPP, UK Biobank Plasma Proteome Project; IVW, Inverse-Variance Weighted Method; FDR, False-Discovery Rate; OR, Odds Ratios; 95 % CI, 95 % Confidence Intervals; PPH4, Posterior Probability of Hypothesis-4; HEIDI, Heterogeneity in Dependent Instruments; GTEx-V8, Genotype-Tissue Expression version-8; GO: Gene Ontology; KEGG, Kyoto Encyclopedia of Genes and Genomes; rQTLs, Ratios QTLs; BMI, Body Mass Index; PPI, Protein-Protein Interaction; FVC, Forced Vital Capacity; FEV1, Forced Expiratory Volume in one second.

## Authors’ contributions

Conceptualization: SLY, CGX, CMS and LJJ; Data curation and Formal analysis: SLY and CGX; Software: CMS; Writing-original draft: CGX and CMS; Visualization: SLY and LJJ; Writing-review & editing: SLY and CMS; All authors read and approved the final manuscript.

## Data availability statement

The current investigation employed publicly accessible GWAS summary statistics as the primary data source. All data are available within the Supplement Table 2.

## Declaration of competing interest

The authors declare no conflicts of interest.

## Appendix Supplementary materials

Image, application 1

## Footnotes

Supplementary material associated with this article can be found, in the online version, at doi:10.1016/j.clinsp.2026.100913.
